# Preliminary estimation of temporal and spatiotemporal dynamic measures of COVID-19 transmission in Thailand

**DOI:** 10.1371/journal.pone.0239645

**Published:** 2020-09-24

**Authors:** Chawarat Rotejanaprasert, Saranath Lawpoolsri, Wirichada Pan-ngum, Richard J. Maude

**Affiliations:** 1 Department of Tropical Hygiene, Faculty of Tropical Medicine, Mahidol University, Bangkok, Thailand; 2 Mahidol-Oxford Tropical Medicine Research Unit, Faculty of Tropical Medicine, Mahidol University, Bangkok, Thailand; 3 Center of Excellence for Biomedical and Public Health Informatics (BIOPHICS), Faculty of Tropical Medicine, Mahidol University, Bangkok, Thailand; 4 Harvard T.H. Chan School of Public Health, Harvard University, Cambridge, Massachusetts, United States of America; 5 Centre for Tropical Medicine and Global Health, Nuffield Department of Medicine, University of Oxford, Oxford, United Kingdom; 6 The Open University, Milton Keynes, United Kingdom; University of Zambia, ZAMBIA

## Abstract

**Background:**

As a new emerging infectious disease pandemic, there is an urgent need to understand the dynamics of COVID-19 in each country to inform planning of emergency measures to contain its spread. It is essential that appropriate disease control activities are planned and implemented in a timely manner. Thailand was one of the first countries outside China to be affected with subsequent importation and domestic spread in most provinces in the country.

**Method:**

A key ingredient to guide planning and implementation of public health measures is a metric of transmissibility which represents the infectiousness of a disease. Ongoing policies can utilize this information to plan appropriately with updated estimates of disease transmissibility. Therefore we present descriptive analyses and preliminary statistical estimation of reproduction numbers over time and space to facilitate disease control activities in Thailand.

**Results:**

The estimated basic reproduction number for COVID-19 during the study ranged from 2.23–5.90, with a mean of 3.75. We also tracked disease dynamics over time using temporal and spatiotemporal reproduction numbers. The results suggest that the outbreak was under control since the middle of April. After the boxing stadium and entertainment venues, the numbers of new cases had increased and spread across the country.

**Discussion:**

Although various scenarios about assumptions were explored in this study, the real situation was difficult to determine given the limited data. More thorough mathematical modelling would be helpful to improve the estimation of transmissibility metrics for emergency preparedness as more epidemiological and clinical information about this new infection becomes available. However, the results can be used to guide interventions directly and to help parameterize models to predict the impact of these interventions.

## Introduction

Two critical pieces of information when a new emerging infectious disease epidemic occurs are the mechanism of disease transmission and how infectious it is. A new emerging and re-emerging infectious disease can occur in one place and have the potential to widely spread, where everyone may be susceptible to the disease. Likewise, in Thailand, COVID-19 caused by the new corona virus is now a major public health concern declared as a national health emergency. In this situation policy makers have to make decisions in the presence of enormous uncertainty and it is crucial to have informed and effective decision making, particularly during the epidemic when the real situation can be very dynamic. During such an outbreak, a large volume of data can be gathered from different sources. These data must be properly transformed into useful and timely information and visualized to effectively assist in the decision-making process. The information should include basic epidemiological descriptions of disease transmission, in terms of person, place, and time. In addition, updated estimates of disease transmissibility should be provided for ongoing planning of appropriate control measures.

For transmitted infections transmitted from human to human such as COVID-19, the spread intensity depends on factors such as the number of infected and susceptible individuals, human contact patterns, and population demographics. Various transmissibility measures can be applied depending on the available data type and can be calculated using different methods. A metric of transmissibility which can be quickly computed is the growth rate, which is estimated from a simple model where disease incidence is exponentially increasing [1]. Another important concept in disease dynamic is the reproduction number, commonly noted as *R*. This quantity measures the expected number of subsequent infections caused by each primary case over the course of their infectious period. The basic reproduction number (*R*_*0*_), probably one of the most widely used of reproduction numbers is defined in a large population where all individuals are susceptible to infection without any control measures. Note that a case here means in a generic sense to represent any infection, even if too mild to meet the clinical case definition [2].

An extensive range of reproduction numbers estimation methods have been proposed (see examples [3, 4]). Although these quantities are helpful to understand the transmissibility of an infectious disease, the estimation methods via transmission models often present difficulties in fitting due to context-specific conditions which usually is unsatisfied [5–7]. However, during the COVID-19 epidemic, disease transmission has been changing rapidly in conjunction with dramatic societal and environmental changes. Therefore, the reproduction number should be updated continually according to these changes. It has been proposed that the temporal variation of an epidemic can be estimated by the temporal (effective) reproduction number over time, *R*_*t*_ [8, 9].

In the WHO report of Thailand COVID-19 situation of 6 February 2020, less than 30 laboratory-confirmed COVID-19 cases were reported by Thai health authorities. Most were visitors to Thailand from China, including Wuhan city in Hubei province. Furthermore, there were 595 suspected cases in Thailand under investigation for COVID-19, while others had been treated for symptoms and discharged. Among the first Thai confirmed COVID-19 cases there were two taxi drivers who possibly had been in contact with infected passengers from China [10]. Since the new coronavirus outbreak began in January 2020 in Bangkok, most newly reported cases were related to transmission clusters including those who had returned from abroad or had occupational exposure to large numbers of people related to businesses in spas, hotels, restaurants, and mall shops. Most cases were male and 20–49 years of age. This was likely because so many cases were linked to boxing stadiums, entertainment venues and to attendance at religious events [11].

COVID-19 was successfully contained in Bangkok for the first two months. However, this was followed by cluster outbreaks in sport and entertainment events, and appearance of the disease in most provinces across the country. Since the contact patterns among individuals differ due to differences in the local environment (e.g. population density, weather), human behavior (e.g. social distancing, working from home, travel), as well as use of personal protection (e.g. facemasks, handwashing), and levels of preexisting immunity, disease transmissibility will vary across locations. The spatial variation of transmissibility between locations should be incorporated to provide more detailed information for policy makers in order to monitor areas at risk. This could potentially be helpful for prioritizing healthcare and public health resources during this outbreak.

Appropriate disease control policies must be planned and implemented in a timely manner. A key ingredient used to guide this public health preparation is the transmissibility metric. This study thus aims to estimate and compare disease dynamic measures in several dimensions that can be augmented with epidemiological summary statistics to monitor the COVID-19 situation for each location and time at different stages of the epidemic. This work can be a complement to current control activities to provide a more complete evaluation of the disease transmission situation in Thailand. As the new coronavirus pandemic continues with changing risk both locally and globally, we hope this work can be a useful addition to the methodological armory to help ongoing planning efforts.

## Materials and methods

### Data sources

The data in this study were from confirmed COVID-19 cases in 77 provinces of Thailand from January 12^th^ 2020 through June 30^th^ 2020 provided in the daily reports of the Department of Disease Control, Thai Ministry of Public Health (MOPH). Suspected cases of COVID-19 infection were identified in hospitals and confirmed at designated laboratories by virus polymerase chain reaction of nose and throat swabs. We collected demographic data and place of diagnosis from the official website developed by the Digital Government Development Agency (https://data.go.th/dataset/covid-19-daily). As all the data used were publicly available, ethical approval was not required.

### Transmission dynamic measures

#### Statistical estimation of reproduction numbers

The first important step to investigate disease transmission is data exploration which combines visualization with calculation of summary epidemiological statistics. Epidemiological measures for infectious diseases include incidence rate, standardized ratios, e.g. standardized incidence ratio or mortality rate (SMR), and cumulative cases. Summary epidemiological statistics are useful information to guide control activities, however these measures are exploratory descriptions that only partially represent disease intensity. Alternatively, the transmissibility of a disease can be referred to the rate where incidence arises in an at-risk population and potentially leads to an outbreak [9, 12–14]. Besides an intrinsic characteristic of an emerging infection, transmissibility can also offer the quantification of disease spread in a given epidemic setting and is impacted by various variables including application of personal protection, contact patterns, and pre-existing immunity status. A range of transmissibility metrics can be adopted suitable for different types of available data and can be approximated using several approaches.

A transmissibility metric which can be quickly calculated is the growth rate. This quantity can be calculated from a basic model where transmission is exponentially increasing and generally defined as the slope of a linear model on logged incidence [1]. During the early stage of an epidemic curve caused by emerging diseases, the exponential growth (EG) rate, denoted by *r*, can be related to the initial reproduction rate and can be described as the change in number of new cases per time unit [15]. Usually calculation of the basic reproduction number requires careful considerations of fundamental conditions to produce precise interpretation. However, in our situation policy makers have to make decisions with the race of disease transmission in the presence of enormous uncertainty. So we aim to make preliminary estimates of the reproduction number with uncertainty intervals under various assumptions for assisting policy makers in this urgent time. Nonetheless, we believe that ongoing investigation and modelling activities should be carried on to assess the effect of public health policies to keep providing updated information to the research community.

As incidence data are non-negative integers, a Poisson likelihood is perhaps suitable to estimate *R*_*0*_, rather than linear Gaussian model of the log scaled of incidence [16]. The exponential growth curve can be used to estimate the reproduction rate [15] and then the basic reproduction number can be estimated as $R_{0}=\frac{1}{M(-r)}$ where *M* is the moment generating function of the serial interval time distribution [15, 17] and *r* is the exponential growth rate during the early stage of an outbreak. Alternatively, the reproduction number can also be calculated using the Maximum Likelihood Estimator (MLE). This method relies on the assumption that the number of secondary cases caused by a primary case follows a Poisson distribution parameterized by *R*_*0*_ in which the likelihood is computed on a period of exponential growth curve [17, 18]. Given the set of observation of incident cases, {***y***_*t*_}, over consecutive time units, *t* = 1,…,*T*, and a generation time distribution over ***w***_*l*_, *R*_*0*_ can be approximated by optimizing the log-likelihood $LL(R_{0})=\sum_{t}^{T}\text{log}(\frac{e^{-\mu_{t}}\mu_{t}^{y_{t}}}{y_{t}!})$ where *μ*_*t*_ is the mean incidence at time *t* and equal to $R_{0}\sum_{l}^{L}y_{t-l}w_{l}$, and *L* is the maximum time for serial interval.

#### Temporal and spatiotemporal transmissibility measures

Even though the basic reproduction number may be useful for understanding the behavior of an emerging disease and designing various intervention strategies, the classic threshold theoretically assumes that the outbreak first occurs in a population with full susceptibility, and hence this quantity is essentially a mathematically defined number and may be less useful in a real disease control situation. It is practically important to assess time-dependent variations in the COVID transmission potential. The pattern of outbreak time series can be partly explained by estimating the effective reproduction number, *R*_*t*_, defined as the expected number of secondary cases per primary case at calendar time *t >* 0 (for examples see [19–21]).

The *R*_*t*_ can represents temporal variation due to the changes in susceptible individuals as intrinsic factors and the operation of control measures which can be seen as extrinsic factors. It suggests that the outbreak is in decline and may be considered as being under control during time *t* when *R*_*t*_ < 1, and, vice versa, if *R*_*t*_ > 1. Several methods to estimate the effective reproduction number exist for emerging diseases [19–21]. Here we adopted on a published method [8, 9, 14] that can be calculated with a stochastic process following the renewal equation in which the series of expected cumulative incidence arise from $Poisson(R_{t}\sum_{l}^{L}y_{t-l}w_{l})$. From this, a data distribution given a set of model parameters can be calculated, as well as the posterior distribution of *R*_*t*_ given collected observations of incidence and knowledge of the serial interval, {***w***_*l*_}[9].

We wanted to provide information that could be used to help design effective control strategies for the current COVID-19 situation in Thailand after the disease has spread to different provinces across the country much of which was from cluster outbreaks originating from several super spreader events. The spatial variation of disease transmission between locations should be incorporated to provide a more detailed picture for policy makers in order to assess areas at risk and can be potentially helpful for prioritizing healthcare and allocation of public health resources during this outbreak. The spatiotemporal reproductive number, *R*_*st*_, for spatial unit *s* at time *t* can be defined as [22] $R_{st}=\frac{\mu_{st}}{\int_{0}^{\infty }w_{l}\mu_{st-l}dl}\approx \frac{\mu_{st}}{\sum_{l=1}^{L}w_{l}\mu_{st-l}}$ where *μ*_*st*_ is the mean incidence in province *s* on day *t*.

To investigate local behavior of disease transmission, we adopted the exceedance probability to identify unusual elevations and detect clusters of COVID-19 incidence. The probability can be estimated by estimating frequency of the measured risk exceeding a threshold and has been used to evaluate how unusual the risk is in an area (see examples [23–26]). Two cutoff thresholds of *R*_*st*_ were chosen as one to represent the null situation (threshold = 1) if the infection was under control and three, a situation where the disease was highly transmitted (threshold = 3) and immediate control policies need to be strictly implemented. Details of transmissibility metrics and parameterization are provided in the supplementary document. Analyses were performed using R (RStudio version 1.2.5001) and WinBUGS [27] software.

## Results

**Fig 1:**
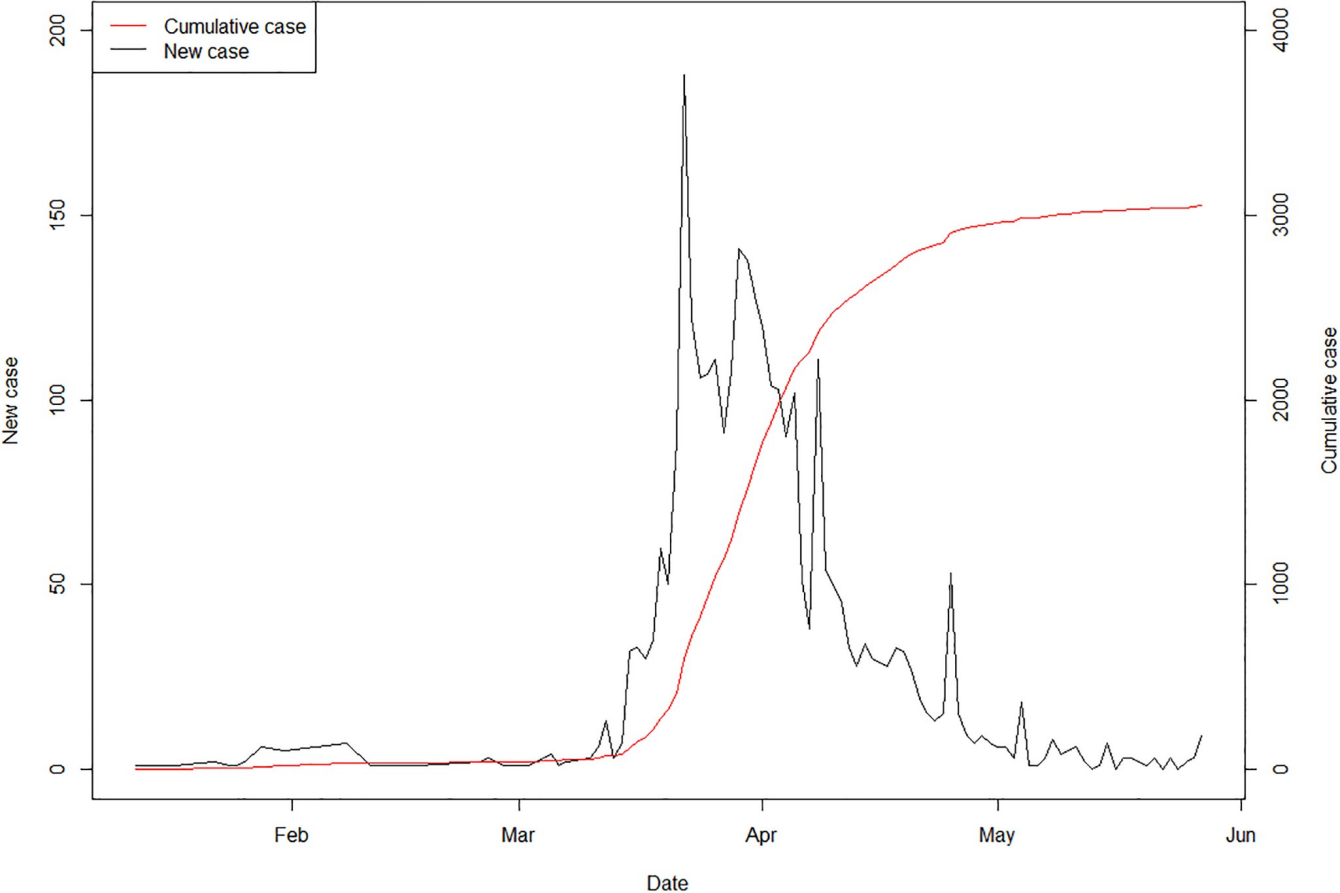
New and cumulative COVID-19 cases in Thailand by report date.

As of June 30^th^ 2020, new and cumulative COVID-19 cases were reported in Thailand as depicted in Fig 1. The left vertical axis shows the number of new COVID-19 cases by day and is represented by the black line. The right vertical axis shows the cumulative COVID-19 total case number represented by the red line. As of June 30^th^ 2020, Figs 2 and 3 show for a 6-day period in mid-March 2020 the daily geographical distributions by province of COVID-19 standardized incidence ratio per 100,000 (calculated as the number of new cases divided by the population at each province), the cumulative number of cases, daily number of new COVID-19 cases and *R*_st_. New and cumulative cases in Fig 1 indicate that the infection had been well controlled until mid-March when the number of cases began to increase. The maps of incidence show that the disease was contained mostly in Bangkok up to mid-March. Following the superspreading events at the entertainment venues and boxing stadium, the distribution of cases then expanded to other provinces outside Bangkok.

**Fig 2:**
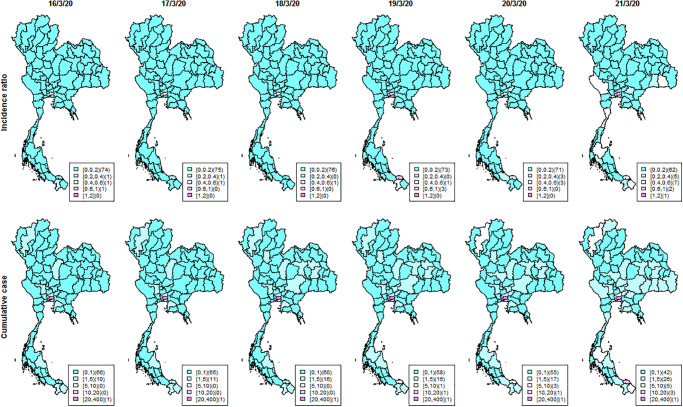
Maps of Thai COVID-19 standardized incidence ratio per 100,000 population and cumulative cases during March 16^th^–March 21^st^ 2020 at provincial level.

**Fig 3:**
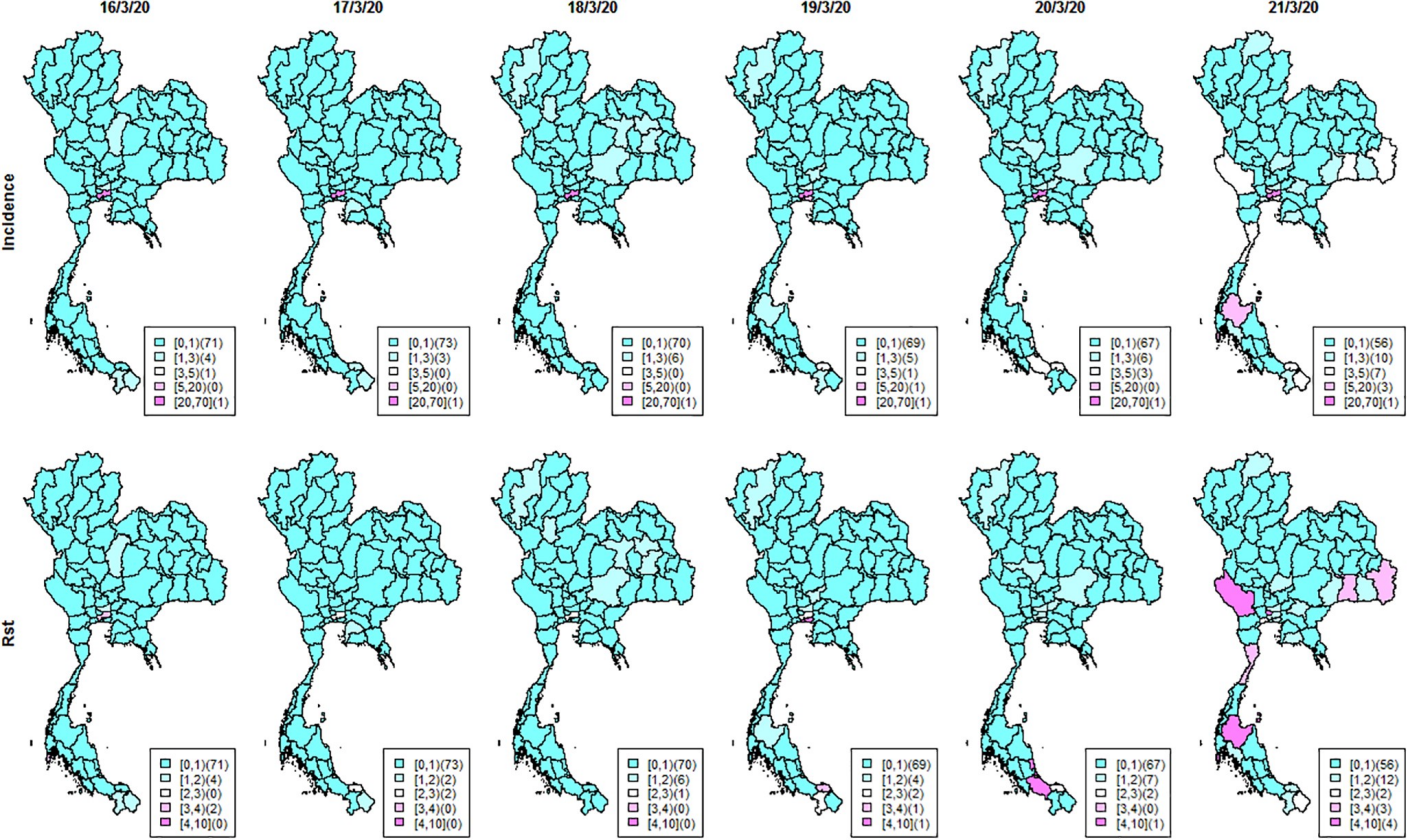
Maps of Thai COVID-19 incidence and *R*_*st*_ during March 16^th^–March 21^st^ 2020 at provincial level.

While we are still learning about this new coronavirus, more epidemiological and clinical information is required now in order to specifically design appropriate control strategies. The transmissibility described by the reproduction number is one of the key ingredients. To approximate the disease dynamics at this early phase we adopted statistical methods to estimate disease transmission measures under various assumptions. Both the exponential growth (EG) rate and maximum likelihood estimation (MLE) were used for estimation of *R*_*0*_ for the COVID-19 situation in Thailand with eleven different studies with parameterized serial intervals in a recent review of available evidence [28]. The estimates were selected from studies with the number of sample pairs larger than twenty. Four of the studies had a pre-print status and seven were published research articles. Most of the estimates were calculated using case information from Asian countries, particularly China. Some of the calculation also applied data from combinations of countries including Italy, Germany and the USA.

Table 1 shows the estimation of *R*_*0*_ for the COVID-19 situation in Thailand with different approximation methods and distributional assumptions of serial interval times. For the EG and MLE methods we also need to supply the time period over which incidence exponentially grow and different time periods can yield very different estimates. The time period from the primary incidence to the date of maximum number of cases could be one option. Nonetheless, because of uncertainty of variation in the early phases of the outbreak, a better choice might be to apply goodness-of-fit criteria to decide the best time period. Since the Poisson distribution was assumed as the data likelihood, the deviance R-squared metric might be suitable to assess over possible time periods. The largest value of the statistics was considered to be the most appropriate period selected for the analysis.

**Table 1:** Estimates of *R*_*0*_ for COVID-19 in Thailand using exponential growth rate (EG) and maximum likelihood (MLE) methods with two different distributional assumptions (Log-Normal and Gamma) of serial interval.

Serial interval (days)	Method	Estimated *R*_*0*_ (95% CI)	
		Log-Normal	Gamma
Mean = 6.3, SD = 4.2 [29]	EG	4.54 (4.09, 5.06)	4.36 (3.96, 4.81)
	MLE	4.44 (3.94, 4.99)	4.31 (3.82, 4.84)
Mean = 4.7, SD = 2.9 [30]	EG	3.46 (3.17, 3.81)	3.42 (3.14, 3.74)
	MLE	3.34 (2.96, 3.75)	3.32 (2.94, 3.73)
Mean = 3.96, SD = 4.75 [31]	EG	2.57 (2.42, 2.75)	2.86 (2.68, 3.06)
	MLE	2.52 (2.23, 2.83)	2.81 (2.49, 3.16)
Mean = 4.4, SD = 3.0 [32]	EG	3.17 (2.92, 3.45)	3.16 (2.92, 3.43)
	MLE	3.06 (2.71, 3.44)	3.08 (2.72, 3.45)
Mean = 5.29, SD = 5.34 [33]	EG	3.22 (2.98, 3.48)	3.34 (3.09, 3.59)
	MLE	3.14 (2.78, 3.53)	3.29 (2.91, 3.69)
Mean = 5.2, SD = 1.72 [34]	EG	4.53 (4.03, 5.12)	4.51 (4.01, 5.09)
	MLE	4.29 (3.80, 4.82)	4.28 (3.79, 4.81)
Mean = 3.95, SD = 1.51 [34]	EG	3.25 (2.97, 3.58)	3.24 (2.96, 3.56)
	MLE	3.07 (2.72, 3.45)	3.06 (2.71, 3.44)
Mean = 6.7, SD = 5.2 [35]	EG	4.49 (4.06, 4.97)	4.28 (3.91, 4.69)
	MLE	4.41 (3.90, 4.95)	4.25 (3.76, 4.77)
Mean = 4.56, SD = 0.95 [36]	EG	4.04 (3.61, 4.55)	4.04 (3.61, 4.54)
	MLE	3.73 (3.31, 4.19)	3.73 (3.30, 4.19)
Mean = 4.22, SD = 0.4 [36]	EG	3.77 (3.38, 4.22)	3.76 (3.38, 4.21)
	MLE	3.26 (2.91, 3.66)	3.26 (2.90, 3.65)
Mean = 7.0, SD = 4.5 [37]	EG	5.25 (4.68, 5.90)	4.95 (4.47, 5.51)
	MLE	5.18 (4.58, 5.82)	4.94 (4.38, 5.45)
Overall	Mean (range)	3.76 (2.23–5.90)	3.74 (2.49–5.51)

Another parameter needed to be defined is the distribution of the serial interval. The empirical distribution could be used from raw data however only the point estimates of mean and standard deviation were reposted in studies and eligible serial-interval distributions are those with positive values. A range of positive-valued distributions were applied to estimate COVID-19 serial interval and the Gamma distribution was commonly used to estimate serial interval times [28]. On the other hand Log-normal modeling also outperformed in a study (both with and without right truncation) [30]. Thus, in this study we assumed the serial intervals to follow Log-normal and Gamma distributions with corresponding parameters for calculation in each scenario. As result, the estimated basic reproduction numbers over our collected data using reasonably exponential curve ranged from 2.23–5.90. The overall means under Log-Normal and Gamma distributions were 3.76 (95% CI: 2.23–5.90) and 3.74 (95% CI: 2.49–5.51) data respectively.

**Fig 4:**
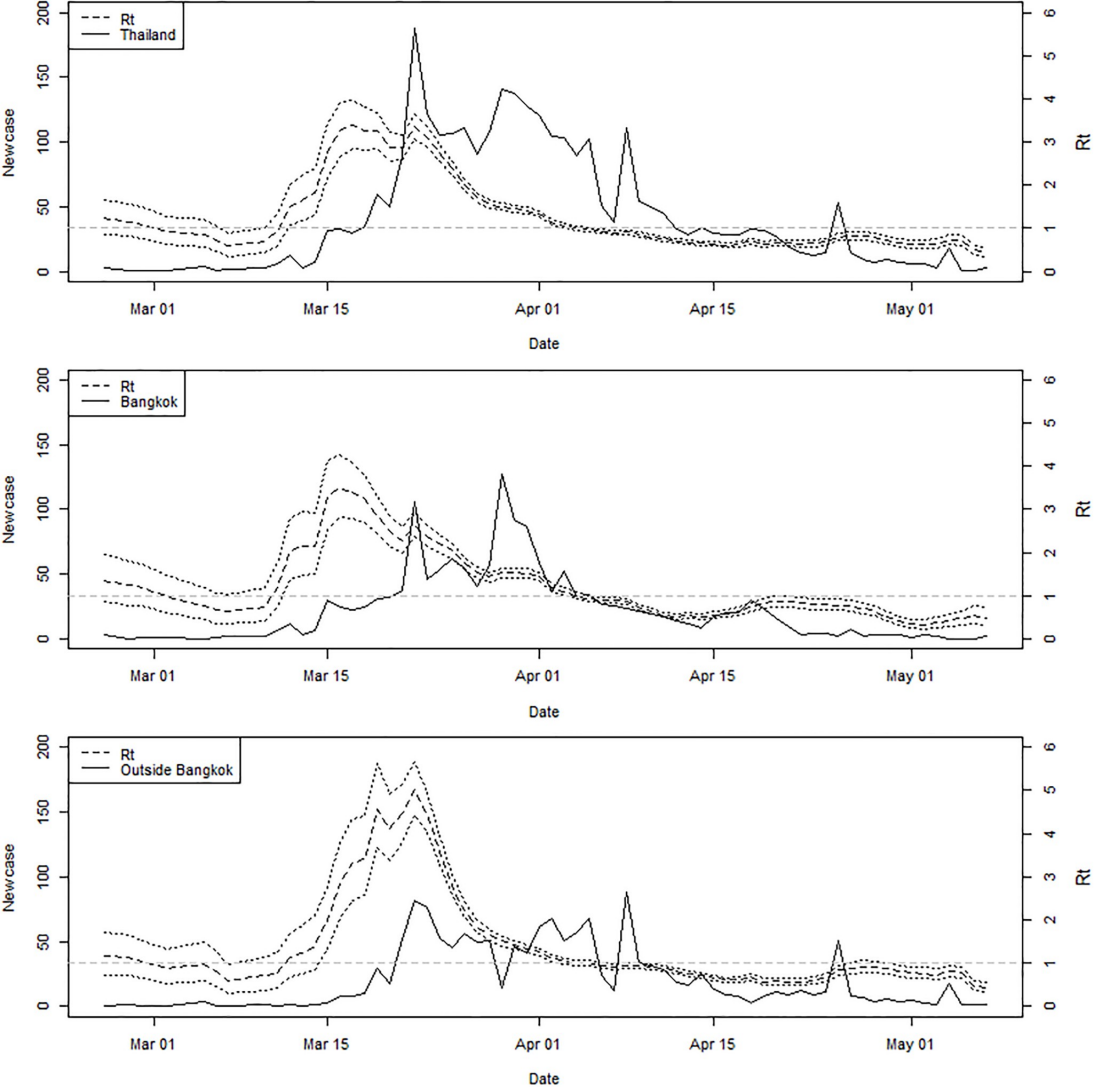
Plots of new cases (solid) and estimated *R*_*t*_ (dash) with 95% CI (dots) in Thailand within and outside Bangkok during February—March 2020. The critical value of *R*_*t*_ = 1 is marked with a grey dashed horizontal line.

Besides the basic reproduction number which merely embodies the disease transmissibility in the whole susceptible population, we also investigated temporal disease variations over time using the effective reproduction number. Fig 4 shows the number of new cases for the whole country (top), Bangkok (middle) and outside Bangkok (bottom) with an estimated *R*_*t*_ (dash) over February-May 2020. The *R*_*t*_ values were approximated by averaging over the serial intervals reported in [30, 36] which were near to the estimated overall mean. Plots in Fig 4 suggest that the outbreak was under control since the middle of April with *R*_*t*_ less than one. After the boxing stadium and entertainment venues, the numbers of new cases had increased until mid-March. Then the number of new cases outside Bangkok sharply increased about one week thereafter with a large jump in *R*_*t*_ while cases in Bangkok started to flatten and then decrease thereafter. The number of new cases continued fluctuating likely due to imported cases returning from overseas. This could be partly related to testing capacity and infection residuals. However the Thai government has implemented travel restrictions including permission to enter or transit Thailand since May. All travelers will be subject to a 14-day state quarantine at a designated facility. So the fluctuation due to traveling might not contribute to local transmission since then.

**Fig 5:**
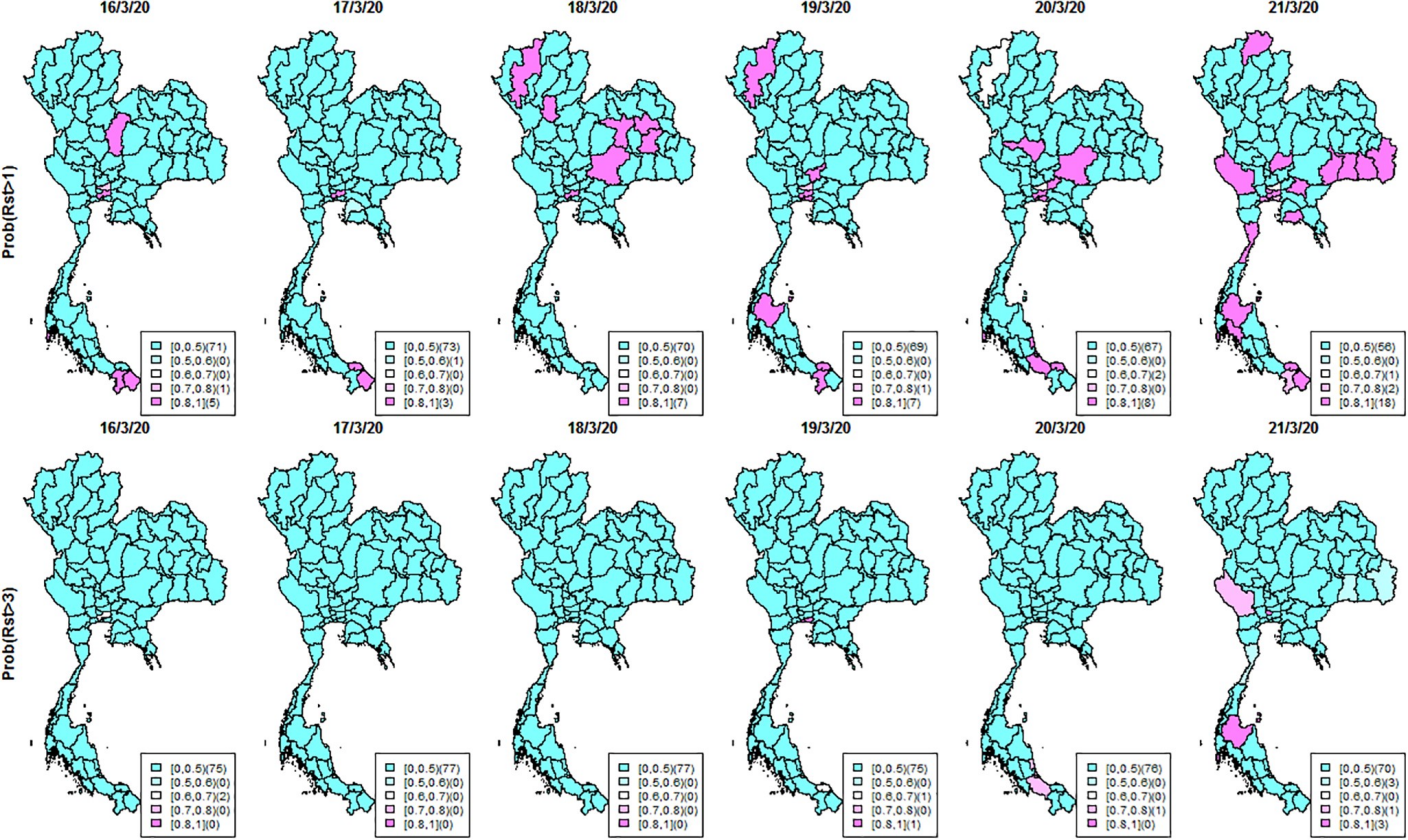
Maps of Thai COVID-19 exceedance probabilities of *R*_*st*_ with thresholds of 1 (top row) and 3 (bottom row) during March 16^th^–March 21^st^ 2020 at provincial level.

The countrywide spread is also reflected in the incidence and *R*_*st*_ maps in Fig 2. Many provinces had few or no cases on March 16^th^. We started to see more cases on March 20^th^, increasing further on March 21^st^ with high *R*_*st*_ in several provinces. To evaluate geographical cluster detection, exceedance probabilities were used as elevated risk diagnostics to investigate geographic patterns of possible spatial anomalies of the infection. In the exceedance maps (Fig 5), we can see only a few scattered provinces with high exceedance probabilities both for thresholds of one (top) and three (bottom) on March 16^th^. However, on March 21^st^ there were increased risks indicated by high exceedance probabilities in the south and west of the country, and some appearing disease clusters spreading over the central region and along the Thai-Cambodia border. This contrasts with the isolated hot spots also found in Northern areas. This method thus helps to identify provinces with more transmission tan others requiring more immediate attention.

## Discussion

When a new emerging infectious disease epidemic occurs, a critical challenge for emergency preparation is that the situation can be very dynamic. Policy makers need to make decisions based on high level of uncertainty. The novel coronavirus infection has been the primary public health concern in Thailand with declaration of a national health emergency. The number of COVID-19 cases has rapidly increased in the country during March 2020. Most cases were initially contained in Bangkok, but this was followed by spread to and transmission in other provinces across the country. Transmission dynamics of COVID-19 in Thailand however remain uncertainly quantified. Thus in this study we present descriptive analyses and quantification of transmission dynamic measures both overall and in space-time dimensions to inform the ongoing control activities of COVID-19 outbreak in Thailand.

Quantifying transmission dynamics of new emerging diseases is a necessary initial step in understanding the epidemic and pandemic potential. We applied statistical methods to available data on cases of novel coronavirus in Thailand to estimate how transmission had varied during February and March, 2020. Preliminary estimation of reproduction numbers was presented with different assumptions to provide a range of possible estimates. Based on available information, we found that the estimate of *R*_*0*_ in Thailand probably varied around 3.75 (95% CI ranging 2.23–5.90) averaged over parameter and distributional assumptions. A number of studies up to early February were reviewed in Liu et al. [38] in which they found that the expected mean *R*_0_ for the infection ranged from 1.4 to 6.49, with a mean of 3.28, a median of 2.79 and interquartile range of 1.16. Another recent systematic review [39] showed the estimated basic reproduction number in China was 2.72 (95% CI: 2.08–3.57) while the estimated outside of China was 4.56 (95% CI: 2.28–9.12). Note that we applied all cases in Thailand available on the website which could include imported cases. However, the sequential cases used to calculate the basic reproduction number during the initial outbreak in Bangkok might be more related to local super-spreader sources such as sport stadiums, entertainment venues and religious events. Our estimates may also be characterized by a number of incidents in which most initial cases locally infected other people. Following initial spread, much of the ongoing transmission was rapidly contained through public health measures including quarantine of infected individuals. Established wide spread transmission from those cases would have caused the transmissibility measures to be considerably higher.

The estimated fluctuations in *R*_*t*_ were driven by the rise and fall in the number of cases, both in Thailand and internationally, as well as prevalence of infection among passengers on evacuation/repatriation flights, other passenger flights having been severely curtailed. Such variations are similar to other studies [40] where causes could include changes in pattern in the population at risk, or specific large spreading events that changed the average transmission estimate. Some evidence of a reduction in *R*_*t*_ was found in the days after the introduction of restrictions in the city of Bangkok, which might have reflected disease control efforts or growing awareness of the infection during that period. There have been no local cases since the end of May and the fluctuation since June was due the imported cases who were subject to a 14-day state quarantine at a designated facility. In this study we applied only publicly available data due to limitations on confidentiality and the uncertainty in our estimates for *R*_*t*_ perhaps was a result from a lack of data availability to inform changes in transmission during the study period. It is also possible that the contact pattern related to infection spread varied over time due to the case variation during the period reflected by effective control strategies implemented against the disease spread. Moreover, the data could include reporting delays which also could limit the performance of *R*_*t*_ in real-time surveillance. However, we have planned to develop a method to correcting for reporting delays which could be beneficial to disease control activities.

The spatiotemporal distribution of cases was supposed to be associated with travel of people who attended entertainment events and Thai boxing in Bangkok in early March. Those people perhaps went home unknowingly carrying the coronavirus. A rise in cases later in the same month may be caused by the implementation of a robust set of public health measures to control the disease situation. This can be seen as the incidence had risen in Bangkok a week after those events followed by the increase in other provinces (Fig 4). The spread might also be related to the government’s lock-down policy in Bangkok. During the same period of time the Bangkok City Hall also imposed the closure of shopping malls, schools, and other venues considered high-risk areas. The workers in those business returned their hometowns and might lead to waves spreading the infection across the country captured in the spatiotemporal transmission dynamics in Figs 1 and 5. The prime minister hence forced a state of emergency across the country, set to be effective until at least the entire month of April 2020. Since then the number of new cases appeared to decline, yet the strict disease control policies have remained in action.

This study aimed to estimate early transmission measures in different dimensions to inform disease control activities. Although several scenarios about assumptions were explored in this study, the real situation was challenging to investigate given the limited data. Our estimation and analysis relied on the information quality of the serial-time interval of the disease, which was highly uncertain especially with Thai data during the study period. In this work, we then employed various generation time scenarios from other studies, some of which also borrowed that information from other similar emerging coronaviruses such as SARS as approximations to that of COVID-19. The improved determination of those parameters is needed and requires further knowledge of the disease transmission chains with a sufficient number of patient subjects and longitudinal times for follow-up [41]. This is unlikely to be achieved shortly especially for local information. More thorough mathematical modelling would be helpful to improve the estimation of transmissibility metrics for emergency preparedness as more epidemiological and clinical information about this new infection becomes available.

## Conclusions

More information is urgently needed as a new emerging infectious disease epidemic evolves presenting a critical challenge for emergency preparation. We present descriptive analyses and preliminary statistical estimation of transmission metrics in over space and time. This new infection has happened in China since the middle of December in 2019, and spread globally with the first case found in Thailand in January 2020. Possible next steps include proposing the most effective control policies to lessen transmission in the country. The working modelling assumptions need to be refined as more is learned about the epidemiological characteristics and outbreak dynamics. Our initial inferences have been made on publicly available data; there perhaps soon be too more information of this new pathogen, and other sources of data should be incorporated.

We believe that ongoing surveillance and modelling efforts should continue to assess the effect of public health measures. The transmissibility estimation should keep going to determine if the transmissibility might vary in different assumptions and regions including socio-economic and climatic contexts. New outbreak clusters of this infection have happened across different countries. As this coronavirus pandemic continues to develop and the risk changes on both local and global scales, hopefully our work can provide an addition to the whole picture of this new infection for research communities and policy-makers.

## Supporting information

S1 File Estimation and evaluation of spatiotemporal disease dynamics. (DOCX)

S2 File R code. (DOCX)
