# Supplementary material for: Preliminary estimation of temporal and spatiotemporal dynamic measures of COVID-19 transmission in Thailand
Source: PLoS One. 2020 Sep 24;15(9):e0239645. doi: 10.1371/journal.pone.0239645 (PMC7514043; doi:10.1371/journal.pone.0239645)
Supplement: S1 File — (DOCX) [file pone.0239645.s001.docx]

**S1 File: Preliminary estimation of temporal and spatiotemporal dynamic measures of COVID-19 transmission in Thailand**

**Estimation of spatiotemporal reproduction number (*R_st_*)**

Let$y_{st}$be the number of new COVID-19 cases at location *s* and time *t* and the disease transmission is presumably modeled with a Poisson process. However, the cases are usually reported at a discrete time such as daily. Assuming the transmissibility remains in the time interval (*t*, *t*+1], the incidence at location *s* time *t* is Poisson distributed with mean$\mu_{st}$. To account for spatiotemporal variation and overdispersion,$\mu_{st}$is modeled to link to a linear predictor consisting of local variables such as environmental and demographic factors and space-time random effects to account for spatiotemporal heterogeneity as $\log(\mu_{st})=\alpha+X_{st}\beta_{st}+u_{s}+v_{s}+\lambda_{t}+\delta_{st}$. The correlated ($u_{s}$) and uncorrelated ($v_{s}$) spatial components have an intrinsic conditional autoregressive (ICAR) prior distribution and zero mean Gaussian distribution respectively. In addition, there are a separate temporal random effect ($\lambda_{t}$) and a space-time interaction term ($\delta_{st}$) in the linear predictor. Often the temporal effect is described using an autoregressive prior distribution allowing for a type of nonparametric temporal effect. Note that this distribution is a random walk prior distribution with one-unit lag. For the interaction term, the prior structure is usually assumed to be distributed as a zero mean Gaussian distribution. The estimate of the reproduction number is however also dependent on the choice of the infectiousness profile or generation time,$w_{l}$, which assumes to follow a Log-Normal distribution standardized to sum to one. Then the full model specification is as follows:

$\begin{matrix} y_{st}\sim Poisson(\mu_{st}) \\ \log(\mu_{st})=\alpha+X_{st}\beta_{st}+u_{s}+v_{s}+\lambda_{t}+\delta_{st} \\ \alpha\sim N(0,\tau_{\alpha}^{-1});\beta_{it}\sim N(0,\tau_{\beta}^{-1}) \\ u_{s}\sim ICAR(\tau_{u}^{-1});v_{s}\sim N(0,\tau_{v}^{-1}) \\ \lambda_{t}\sim N(\lambda_{t-1},\tau_{\lambda}^{-1}) \\ \delta_{st}\sim N(0,\tau_{\delta}^{-1}) \end{matrix}$ $\begin{matrix} R_{st}=\frac{\mu_{it}}{\sum_{l=1}^{L} w_{l}\mu_{st-l}} \\ w_{l}=\frac{\exp(g_{l})}{\sum_{l=1}^{L} \exp(g_{l})} \\ g_{l}\sim Normal(0,\tau_{g}^{-1}) \\ \tau_{*}^{-1/2}\sim Unif(0,10). \end{matrix}$ (1)

**Evaluation of spatiotemporal adverse risks using exceedance probability**

To investigate localized behavior of COVID-19 case clusters, an exceedance probability is an important tool for the assessment of unusual elevation of disease. The probability can be estimated by recording how often the risk exceeds a threshold and has been used to evaluate how unusual the risk is in an area. Here we would like to explore the COVID-19 risk at each location and time interval. Then the spatiotemporal reproduction number is appropriate in this case. The exceedance probability for *R_st_* is usually calculated from the posterior sample values and defined as

$P(R_{st}>c)=\sum_{i}^{I} \frac{1(R_{i,st}>c)}{I}$ (3)

where *I* is the posterior sampler sample size and 1(.) is the indicator function. There is another component to be considered for the exceedance probability to be employed: the cut-off point *c* for *R_st_*. The threshold *c* for an exceedance probability is referred to as the level used to define an area with an unusual risk. It suggests that the outbreak is in decline and may be regarded as being under control during time *t* when *R_st_* < 1, and, vice versa, if *R_st_* > 1. So the cut-off threshold should be *c* = 1 for the null value. However, we would also like to consider extreme situations where disease control policies need to be implemented urgently. So another value of *c* = 3 is considered. That is to assess possible spatial adverse risk in each location we calculate $P(R_{st}>c)$ with *c* representing a range of extreme risks with 1 as the benchmark and 3 for extreme anomaly.
